# Identification of passive wrist-worn accelerometry outcomes for improved disease monitoring and trial design in motor neuron disease

**DOI:** 10.1016/j.ebiom.2025.105779

**Published:** 2025-05-29

**Authors:** Cory J. Holdom, Rakesh Pilkar, Christine C. Guo, Ruben P.A van Eijk, Nadia Sethi, Robert D. Henderson, Shyuan T. Ngo, Frederik J. Steyn

**Affiliations:** a Australian Institute for Bioengineering and Nanotechnology, The University of Queensland, Australia; b ActiGraph, LLC, Pensacola, FL, USA; c Department of Neurology, UMC Utrecht Brain Center, University Medical Center Utrecht, the Netherlands; d Biostatistics & Research Support, Julius Center for Health Sciences and Primary Care, University Medical Center Utrecht, Utrecht, the Netherlands; e Clinical Pharmacogenomics and Precision Medicine, University of Florida, FL, USA; f Centre for Clinical Research, The University of Queensland, Australia; g Department of Neurology, Royal Brisbane and Women's Hospital, Brisbane, Australia; h School of Biomedical Sciences, The University of Queensland, Australia

**Keywords:** Amyotrophic lateral sclerosis, Clinical outcome measures, Actigraphy, Remote monitoring

## Abstract

**Background:**

Motor neuron disease (MND) leads to progressive functional decline, making reliable measures of disease progression critical for patient care and clinical trials. Current clinical outcome measures lack the ability to continuously and objectively track functional decline in daily life of patients with MND. This study assessed and validated wrist-worn accelerometry outcome measures for continuous monitoring in MND, with the potential to refine clinical trial outcomes.

**Methods:**

This longitudinal study included 95 patients with MND who wore an ActiGraph GT9X Link device on their non-dominant wrist for 8 days, with follow-up every 3–4 months. Accelerometer data were processed using ActiLife and GGIR. Joint models were used to simultaneously investigate the longitudinal change in ALS Functional Rating Scale-Revised (ALSFRS-R) scores and accelerometer-derived outcomes alongside their relationship with overall survival. Sample size estimates for clinical trials were generated using both accelerometer- and ALSFRS-R-based outcomes, and principal component analysis (PCA) explored outcome relationships.

**Findings:**

Accelerometer outcomes showed a slower rate of decline (−0.03 to −0.07 SD/month) compared to ALSFRS-R (−0.10 SD/month) and had stronger correlations with ALSFRS-R motor subdomains (partial r: 0.60–0.73). PCA revealed that longitudinal measures of accelerometry were distinct from the ALSFRS-R, highlighting the complementary nature of these measures. Peak 6-min activity predicted smaller clinical trial sample sizes for studies over 12 months. Accelerometer-derived outcomes were not significantly associated with survival.

**Interpretation:**

Wrist-worn accelerometry offers a practical solution for continuous monitoring in MND, complementing ALSFRS-R. Measures of peak performance, and specifically peak 6-min activity shows promise, potentially reducing sample sizes and improving disease tracking over longer duration studies. Further refinement and validation are needed to adopt actigraphy measures as clinical assessment outcomes.

**Funding:**

This study was supported by 10.13039/100011890Wesley Medical Research (2016-32), the Honda Foundation, 10.13039/100016887Motor Neurone Disease Research Australia, and 10.13039/100014012FightMND. CJH received a Higher Degree Research Scholarship from UQ. STN received support from the Scott Sullivan Fellowship (MND and Me Foundation/10.13039/501100001077RBWH Foundation), a 10.13039/100014012FightMND Mid-Career Fellowship, and the 10.13039/501100024735AIBN.

**Research in context**

**Evidence before this study**

We searched PubMed and relevant clinical trial databases for studies investigating the use of wrist-worn accelerometers in the monitoring of disease progression in amyotrophic lateral sclerosis (ALS) and motor neuron disease (MND). The search included terms “accelerometry”, “actigraphy” and “ALS” or “MND”, with no language restrictions. Studies published before October 2024 were included. The existing literature suggests that accelerometry can capture motor function and track disease progression in neurodegenerative diseases, but most previous studies focused on general activity levels, with limited focus on wrist-worn accelerometers. While wrist-based devices have been shown to provide granular data, their application in clinical trials for MND has been underexplored. Additionally, no study has systematically evaluated which wrist-based measures are most sensitive and clinically meaningful for tracking functional decline in patients with MND over time.

**Added value of this study**

This study provides robust evidence supporting the use of passive, wrist-worn accelerometers to monitor disease progression in MND. Measures of movement, including measures of maximal movement, demonstrate functional decline. Our findings also show that wrist-based accelerometry can reduce the sample size required for longer-term clinical trials and provide complementary insights into motor function, offering a scalable approach to enhance disease monitoring in clinical settings.

**Implications of all the available evidence**

Our findings have important implications for both clinical practice and trial design. By integrating passive wrist-worn accelerometers into clinical trials, researchers and clinicians can monitor disease progression, reduce patient burden, and optimise trial efficiency. The insights gained from this study can be applied not only to MND but potentially to other neuromotor diseases, expanding the scope of wearable technology in clinical research. Further development and refinement of wrist-based metrics may improve the real-world applicability of remote monitoring tools.

## Introduction

Motor Neuron Disease (MND) is a group of neurodegenerative diseases characterised by the progressive loss of motor neurons in the cortex, medulla, and/or spinal cord.^1^ Associated symptoms include progressive weakness, spasticity, and atrophy in the associated motor unit, leading to eventual paralysis. Amyotrophic Lateral Sclerosis (ALS) is the most common form of MND, with a median survival of 2–5 years following disease onset.^1^ There are few effective treatments for ALS. The most effective treatment for the majority of ALS cases thus far is riluzole, providing an estimated extension of 6 months survival,^2^^,^^3^ with benefit possibly even in people with late-stage ALS.^4^ The development and implementation of new treatments in ALS is hampered, in part, by the lack of effective clinical outcome measures that accurately capture the functional decline of people living with ALS outside of a traditional clinical setting.^5^

Actigraphy, which leverages wearable sensors to monitor movement, provides a promising platform for identifying novel outcome measures outside the clinic. By continuously capturing real-world functional abilities, wearable devices like wrist-worn accelerometers may offer a more accurate and comprehensive assessment of disease progression in ALS, complementing traditional clinical evaluations. This approach could improve the sensitivity of outcome measures in clinical trials and potentially accelerate the development of new treatments. While actigraphy has been proposed as a secondary outcome for clinical trials,^6^, ^7^, ^8^ there is no consensus on what features should be assessed to effectively monitor disease progression. Previous studies have demonstrated that vertical variability measures from hip-worn trackers can monitor gross physical changes and have potential to power future studies.^7^^,^^9^ However, our investigations found that these specific hip-derived outcomes were less effective when applied to the wrist-worn devices,^10^ indicating the need for the development and validation of wrist-specific measures of disease progression.

Here, we explore the use of non-dominant wrist-based accelerometry combined with publicly available algorithms to monitor disease progression in patients with MND. Our findings show that careful selection of output measures can enhance the ability of wrist-based actigraphy to track disease progression. Measures of maximal function appear most effective in reducing the number of participants needed to meet clinical trial outcomes over longer durations. While current algorithms may have limitations in short-term monitoring, these measures offer complementary insights and highlight the need for further refinement of remote monitoring tools to optimise their use in clinical trials.

## Methods

### Study design and clinical assessment

**Fig. 1:**
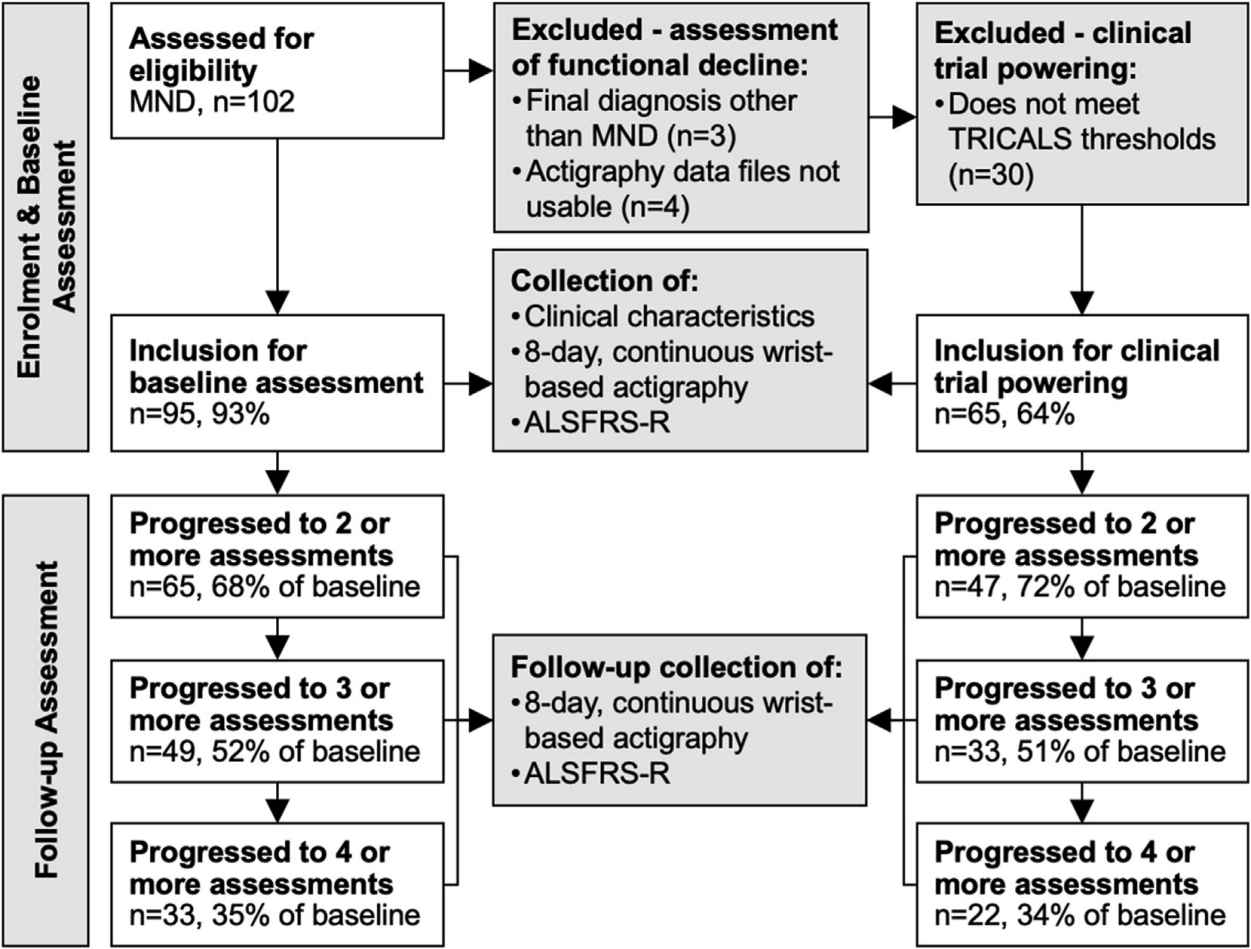
**Enrolment schematic summarising number of patients within the study over time**.

This longitudinal natural history study is based on data collected between 28 February 2017 and 15 September 2021. Patients (n = 102) who attended the Royal Brisbane and Women's Hospital (RBWH) MND clinic who received a diagnosis of MND were invited to participate. Participants disclosed their sex at study enrolment. We did not ask participants to disclose their race or ethnicity. Sex, race and ethnicity were not used as an inclusion/exclusion factor. Patients with a diagnosis of frontotemporal dementia were not included in this study. Enrolment details are shown in Fig. 1. Diagnosis was determined based on the Gold Coast criteria.^11^ Patients with any form of adult-onset MND were included in the analysis; three participants with a final diagnosis of PLS were included in our analysis of whole cohort data. Seven participants were excluded from the final analysis of functional decline, three due to receiving a final diagnosis other than definite MND, four due to incomplete actigraphy data recordings. To investigate potential clinical trial outcomes, we conducted a further analysis limiting inclusion to participants meeting a Treatment Research Initiative to Cure ALS (TRICALS) risk profile^12^ between −6 and −2 (both inclusive) eligibility window (mirroring the thresholds implemented in current ALS clinical trials—e.g., NCT06008249); thirty participants were excluded. All participants meeting the TRICALS criteria had a final diagnosis of ALS. Participants were invited to return for subsequent research visits every ∼3 months (median latency between visits = 3.43 months for participants included in the assessment of functional decline, and 3.42 for those included in clinical trial powering). For the assessment of functional decline, the median number of visits per participant was 3.00, for a total of 289 participant-visits and a total of 863.03 participant-months of monitoring. For the assessment of clinical trial powering, the median number of visits per participant was 3.00, for a total of 200 participant-visits and a total of 596.11 participant-months of monitoring.

### Ethics

This study was approved by The University of Queensland (2015/HE000022), RBWH (HREC/14/QRBW/495), and Wesley Hospital (1622) human research ethics committees. All participants provided written, informed consent.

### Anthropometric and clinical measures

Participants’ height was measured with a stadiometer, and their mass was measured with the BODPOD Gold Standard system (COSMED, Concord, CA, USA) for initiation of the *ActiGraph* GT9X Link devices (ActiGraph, L.L.C., Pensacola, FL, USA). Body mass index was calculated as body mass divided by the square of their height. The clinical history of participants was recorded, and the ALS Functional Rating Scale-Revised (ALSFRS-R)^13^ was collected in-person by a member of the research team, or from existing clinical records provided by the RBWH. The ALSFRS-R was summated into four domains: “bulbar” (questions 1–3), “upper-limb” (questions 4–5), “lower-limb” (questions 8–9), “respiratory” (questions 10–12), and a combined “motor” domain (questions 4–9). Responses to all ALSFRS-R questions are presented in Supplemental Table S1. The slope of the ALSFRS-R with respect to time (ΔFRS) was defined as [ΔFRS = (ALSFRS-R score–48)/months since symptom onset].

### Initiation of actigraphy devices

Participants were provided an *ActiGraph* GT9X Link device to wear on their non-dominant wrist continuously for 8 consecutive days, with the recording beginning at 11:59 AM of the day the device was given. Participants were prompted to remove the device when instructed (following the appearance of a “stop hand” on the screen after the 8 days) and were asked to inform the research team if they were not able to complete the recording due to discomfort or device failure. All patients were asked to wear the device, regardless of their level of upper-limb disability. Carers or family members were instructed on proper device placement and were asked to assist if the device was adjusted due to discomfort. Importantly, we did not exclude participants based on severe limb impairment. Devices were initialised with the ActiLife software (version 6.13.4) and the GT9X was set up to record tri-axial accelerations at 30 Hz. Upon return of the GT9X, raw accelerometer, as well as summarised “count” recordings (10s epochs) were downloaded using the ActiLife software. The “low frequency extension” setting^14^ was enabled to increase sensitivity for subtle movements.

### Accelerometer scoring

Raw accelerometer recordings were scored using the GGIR package (version 2.8–2) in R (version 4.2.2) with default settings. From this, we derived the average Euclidean norm minus one (ENMO), as well as time spent in medium-to-vigorous physical activity (MVPA). 5-second epochs with a ENMO above 100 were considered active. Additionally, the peak 5-h ENMO was included as an outcome. The “Verisense” algorithm was used to estimate step counts.^15^ Additionally, “activity count data” from ActiLife were used to derive step count estimates (based on an algorithm tuned for hip devices) and estimate the 95th percentile (“Peak”) daily activity from open source ActiGraph activity counts^16^ within a sliding 6-min window. This combination of features represents both people's average and maximal daily activities. The 12 h ends of each recording were trimmed to allow for a 7-day monitoring period and the week-long average of each daily summary was used for downstream analyses. Supplemental Table S2 summarises each feature considered for the analysis. Where features were available with multiple epochs, we included features with the fastest rate of decline over time (Supplemental Table S3).

### Sample size estimation

While previous studies have used wrist-worn devices to monitor physical activity in ALS, none have reported sample size considerations based on peak performance metrics such as peak 6-min activity. However, since related accelerometry-derived outcomes (e.g., vector magnitude inactivity, VMI) have been shown to correlate strongly with ALSFRS-R total scores,^7^ we based our sample size considerations on the established relationship between ALSFRS-R and overall survival. We assumed that each one-point increase in ALSFRS-R total score is associated with a 12% reduction in the hazard of death (HR = 0.88), and that the standard deviation of ALSFRS-R at baseline is 5 points.^17^ Under these assumptions, 30 events provide 92% power to detect the hypothesised hazard ratio using a log-rank test with a two-sided alpha of 0.05.

### Statistics

Data were reported as mean (SD), median [quartile 1, quartile 3], or n (%). We used a joint model to quantify the change in the outcomes of interest (accelerometer-derived outcomes and ALSFRS-R) over time and explored the relationship between these longitudinal outcomes and their influence on survival. Participants were censored after 36 months to reduce sensitivity to outliers from participants with extended study participation. Outcomes are reported in their base units as well as standard deviations per month. The mixed effects component of the model included a participant-varying random slope for time and intercept, and the survival sub-model included age at study enrolment and sex as covariates following a Weibull baseline hazard. To estimate correlations between the ALSFRS-R and accelerometer-derived outcomes, we randomly selected one collection from each participant (total: 95 datapoints per correlation) and conducted Pearson correlations. For the correlational analyses, Holm correction was used to inflate p values for multiple comparisons. The joint models were additionally used to estimate hazard ratios for each longitudinal outcome with survival outcomes. The origin of survival time was defined as the time (in months) of first accelerometry data collection for each participant. Hazard ratios (HRs) are reported as estimate [95% CI]. For illustrative purposes, Kaplan–Meier survival curves were created by grouping based on their initial ALSFRS-R Motor score or “Peak” activity being above/below the cohort median, and by ΔFRS being above or below −0.5 month^−1^ at the initial research visit. Significance for metrics measured at baseline with overall survival curves were estimated from log-rank tests. Bootstrap resampling with replacement was used to estimate trial arm sizes for a theoretical clinical trial based on our estimated effect sizes. As has been done previously,^7^ we considered a trial with a 30% improvement in the outcome measure and a power of 80% to estimate the number of participants per arm required to detect this treatment effect, given week-long, 3-monthly assessments.

All data analyses were prepared in RStudio (version 2022.12.0 + 353; Posit Software, 2022) running R (version 4.2.2; The R Foundation for Statistical Computing Platform, 2022). The GGIR^18^ (version 2.8–2), lme4^19^ (version 1.1–30) and performance^20^ (version 0.10.2) packages were used for analyses. ggplot^21^ (version 3.4.0), and ggpubr^22^ (version 0.4.0) packages, as well as Microsoft PowerPoint (version 2004) were used for data presentation.

### Role of funders

Funders had no role in study design, data collection, data analyses, interpretation, or writing of this report.

## Results

### Passive accelerometer-derived outcomes decline over time alongside ALSFRS-R

**Fig. 2:**
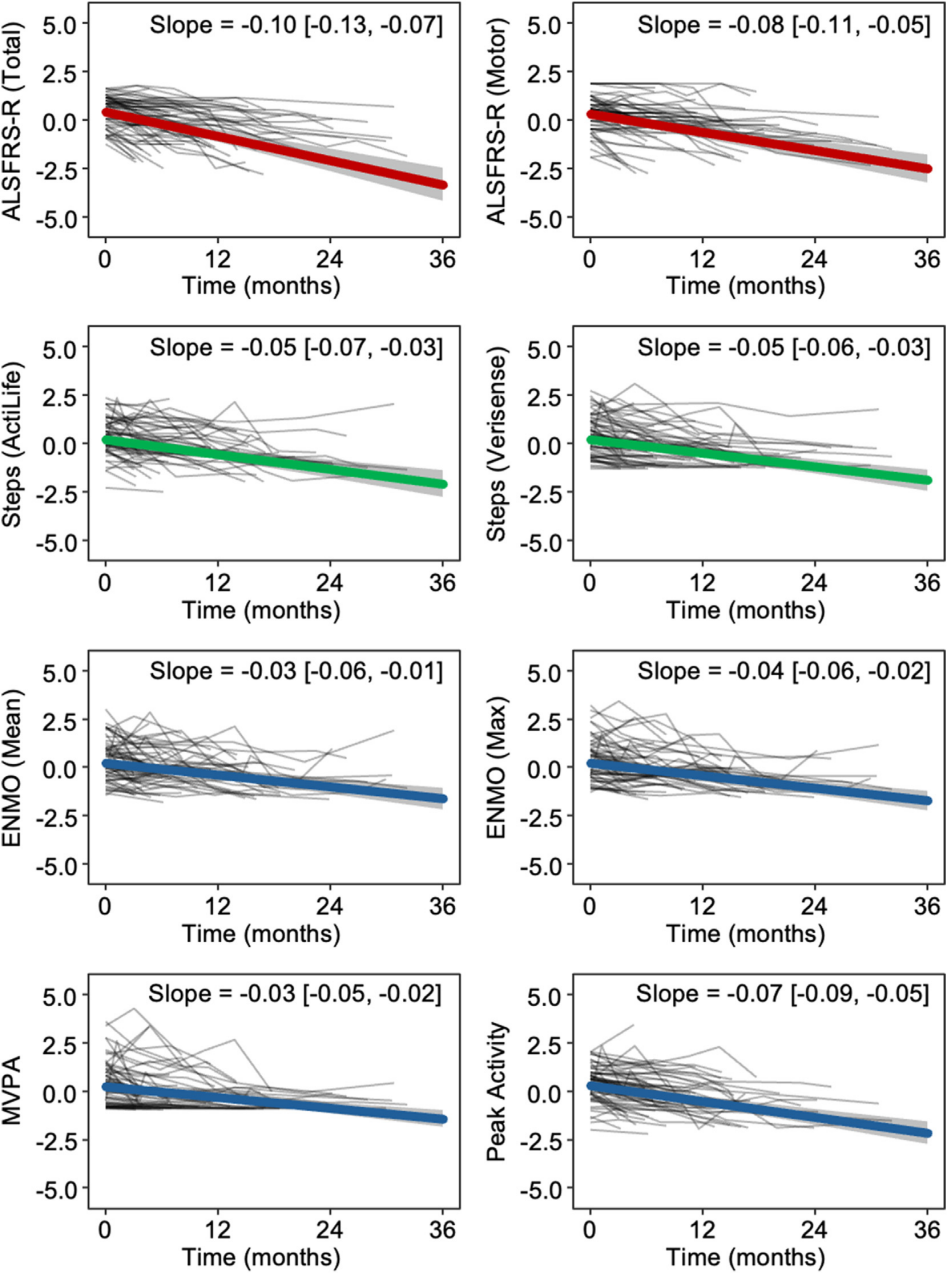
**Spaghetti plots illustrating changes in accelerometer-derived outcomes and ALSFRS-R over time**. Outcomes are standardised to standard deviations from the mean (n = 95). Black lines show per-participant individual traces over time, and coloured line shows a “population” trend. Coloured trendline estimated from the mixed-effects component of a joint model, with a random effect for time and participant. Age and sex were considered covariates for survival. 95% CI of the estimate is shaded in grey. All estimates are p < 0.0001. ALSFRS-R, amyotrophic lateral sclerosis functional rating scale—revised; ENMO, Euclidean norm minus one; MVPA, time spent in moderate to vigorous activity.

Demographics, clinical characteristics, and specifics regarding actigraphy are presented in Table 1. 22/95 (23%) participants were female, due to the higher prevalence of MND in males. The trajectories of accelerometer-derived outcomes are summarised in Table 2. Whole cohort data are visualised in Fig. 2, and data for participants who met TRICALS criteria are presented in Supplemental Figure S1. For direct comparability, outcomes are normalised by subtracting the mean and scaling by their SD. There was a faster rate of decline in the ALSFRS-R (total: −0.10; motor: −0.08 SD/month) when compared to the accelerometer-derived outcomes (−0.6 to −0.3 SD/month). Notably, the rate of decline in peak 6-min activity (−0.07 SD/month) was comparable to the ALSFRS-R motor subdomain (−0.08 SD/month), supporting its potential relevance as a marker of functional change. Rates of decline for most measures were slightly steeper for participants who met TRICALS criteria.

**Table 1:** Characteristics of all study participants (whole cohort), and participants who met the TRICALS risk profile for inclusion in clinical trials.

	Whole cohort (n = 95)	Clinical trial cohort (n = 65)
**Demographics**		
Female, n (%)	22 (23%)	16 (25%)
Age, years	59.95 ± 9.32	61.90 ± 8.74
Body-mass index, kg m^−2^	26.43 ± 4.75	25.95 ± 4.27
**Clinical measures**		
Bulbar onset, n (%)	25 (26%)	21 (32%)
Age at onset, years	57.85 ± 9.40	60.19 ± 8.49
Diagnostic delay, months	11.0 [7.0, 18.0]	10.0 [6.0, 13.0]
Time since onset, months	20.0 [14.0, 27.0]	19.0 [14.0, 23.0]
ALSFRS-R	37.38 ± 5.47	37.22 ± 5.26
Bulbar sub-score (Q1–3)	9.92 ± 2.00	9.80 ± 2.12
Upper-limb sub-score (Q4–5)	5.89 ± 2.08	5.98 ± 2.01
Lower-limb sub-score (Q8–9)	4.70 ± 2.15	4.66 ± 2.16
Motor sub-score (Q4–9)	16.44 ± 4.56	16.45 ± 4.61
Respiratory sub-score (Q10–12)	11.02 ± 1.52	10.97 ± 1.57
ΔFRS, month^−1^	−0.42 [−1.00, 0.00]	−0.52 [−1.00, 0.00]
FVC, % of predicted	86.20 ± 18.02	83.74 ± 18.84
Concomitant dementia diagnosis	0 (0%)	0 (0%)
TRICALS risk score	−4.44 [−5.41, −3.67]	−4.16 [−4.81, −3.51]
**Actigraphy**		
Number of recordings/participant	3.0 [1.0, 5.0]	3.0 [1.0, 40]
Interval between recordings, months	3.43 [3.07, 4.40]	3.42 [3.02, 4.34]

Demographic and clinical features of participants at time of enrolment. Sex and site of onset reported as n (%); age, body-mass index, ALSFRS-R and FVC reported as mean ± standard deviation; other values reported as median [quartile 1, quartile 3]. ALSFRS-R, Amyotrophic Lateral Sclerosis Functional Rating Scale–Revised; ΔFRS, average monthly decline in ALSFRS-R since symptom onset; FVC, seated force vital capacity; TRICALS, Treatment Research Initiative to Cure ALS.

**Table 2:** Per-month change in accelerometer outcomes and ALSFRS-R for the whole cohort, and participants who met the TRICALS risk profile for inclusion in clinical trials.

	Whole cohort (n = 95)				Clinical trial cohort (n = 65)			
	Slope (absolute)		Slope (SD/month)		Slope (absolute)		Slope (SD/month)	
ALSFRS-R (Total)	−0.62	[−0.81, −0.43]	−0.10	[−0.13, −0.07]	−0.73	[−0.99, −0.48]	−0.11	[−0.15, −0.07]
ALSFRS-R (Motor)	−0.38	[−0.51, −0.25]	−0.08	[−0.11, −0.05]	−0.45	[−0.62, −0.29]	−0.09	[−0.12, −0.06]
Steps (ActiLife)	−293	[−416, −169]	−0.05	[−0.07, −0.03]	−353	[−353, −353]	−0.06	[−0.09, −0.03]
Steps (Verisense)	−145	[−203, −86]	−0.05	[−0.06, −0.03]	−166	[−251, −82]	−0.05	[−0.08, −0.03]
ENMO	−0.26	[−0.42, −0.10]	−0.03	[−0.06, −0.01]	−0.28	[−0.50, −0.06]	−0.04	[−0.07, −0.01]
ENMO (5 h Peak)	−0.66	[−1.00, −0.31]	−0.04	[−0.06, −0.02]	−0.71	[−1.17, −0.26]	−0.04	[−0.07, −0.02]
MVPA (5s epoch)	−1.10	[−1.59, −0.61]	−0.03	[−0.05, −0.02]	−1.22	[−1.90, −0.55]	−0.04	[−0.06, −0.02]
Peak 6 min activity	−17.38	[−21.81, −12.96]	−0.07	[−0.09, −0.05]	−19.92	[−25.75, −14.08]	−0.08	[−0.10, −0.06]

Estimates are derived from joint models, with a time-varying random slope and intercept for participant. Survival component of models include age and sex as covariates. Values are shown as estimate [95% CI]. All estimates are p < 0.0001. ALSFRS-R, amyotrophic lateral sclerosis functional rating scale—revised; ENMO, Euclidean norm minus one; MVPA, time spent in moderate to vigorous activity; TRICALS, Treatment Research Initiative to Cure ALS.

**Fig. 3:**
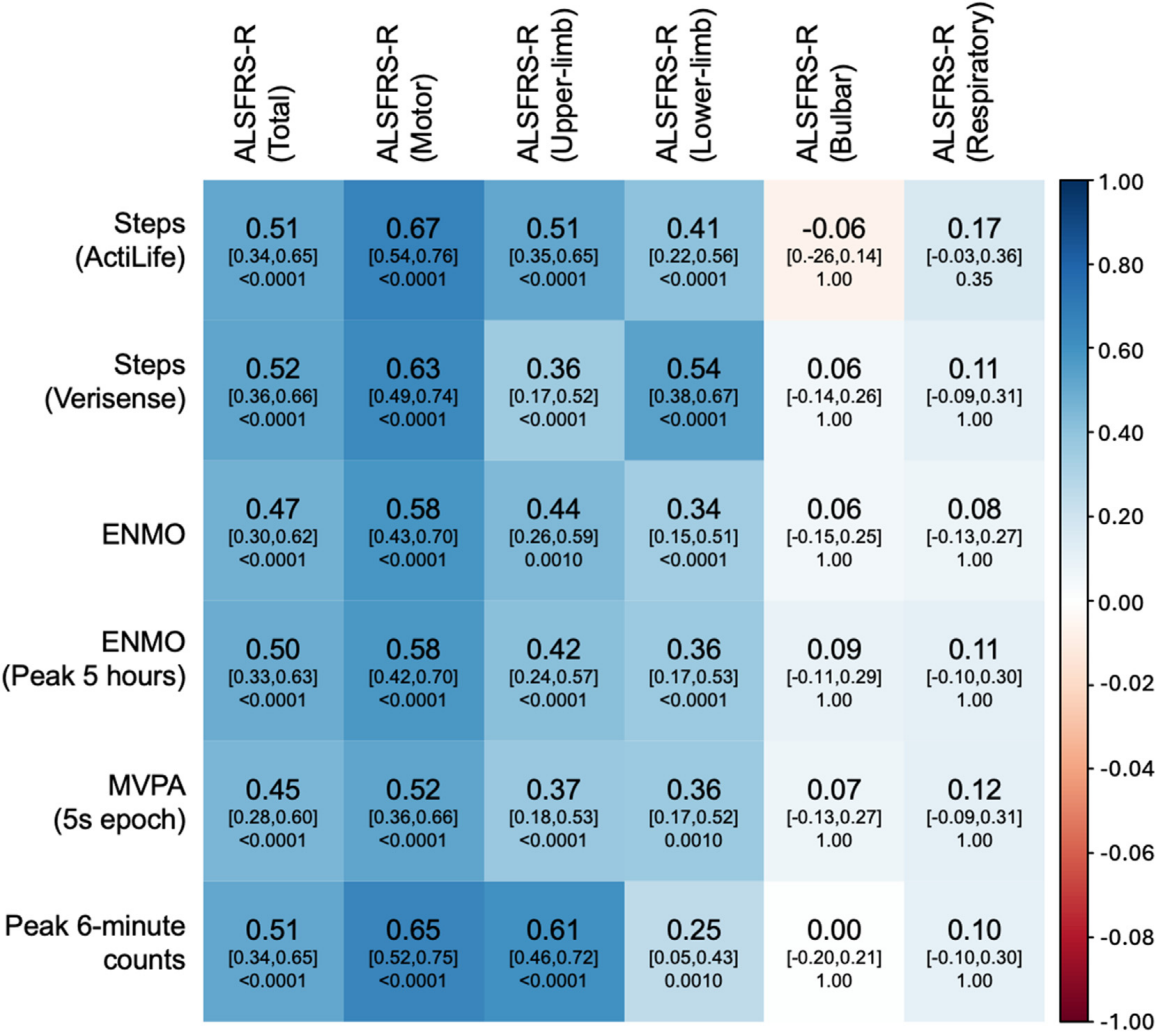
**Partial Pearson correlation matrix (adjusted for “time”).** Correlations were estimated from the per-participant residuals from a joint model, with regressions fit to each parameter over time to control for time as a confounding factor (n = 95). Age and sex were considered covariates for survival. Correlation strengths are shown within the matrix as numeric and colour. Main numbers show estimated correlation coefficients; subscripts show p values. ALSFRS-R, amyotrophic lateral sclerosis functional rating scale—revised; ENMO, Euclidean norm minus one; MVPA, time spent in moderate to vigorous activity.

Fig. 3 presents the outcomes of the correlations between the ALSFRS-R, and the accelerometer-derived outcomes. The accelerometer-derived features showed low–moderate correlations (range: 0.39–0.47) with total ALSFRS-R. The strength of these correlations increased when limiting analysis to ALSFRS-R scores within the motor domain (range: 0.48–0.65), suggesting that most of the association with ALSFRS-R was driven by changes in the motor domain alone. When comparing upper vs lower limb scores, stronger correlations were seen with the “upper-limb” scores (range: 0.38–0.62) than the “lower-limb” scores (range: 0.15–0.48). Weak associations were seen with the non-limb domains (bulbar range: −0.03 to 0.08; respiratory range: −0.03 to 0.06, all nonsignificant). Similar outcomes were seen when limiting the analysis to participants who met TRICALS criteria (Supplemental Figure S2).

### Accelerometer-derived outcomes capture a different aspect of MND compared to the ALSFRS-R

**Fig. 4:**
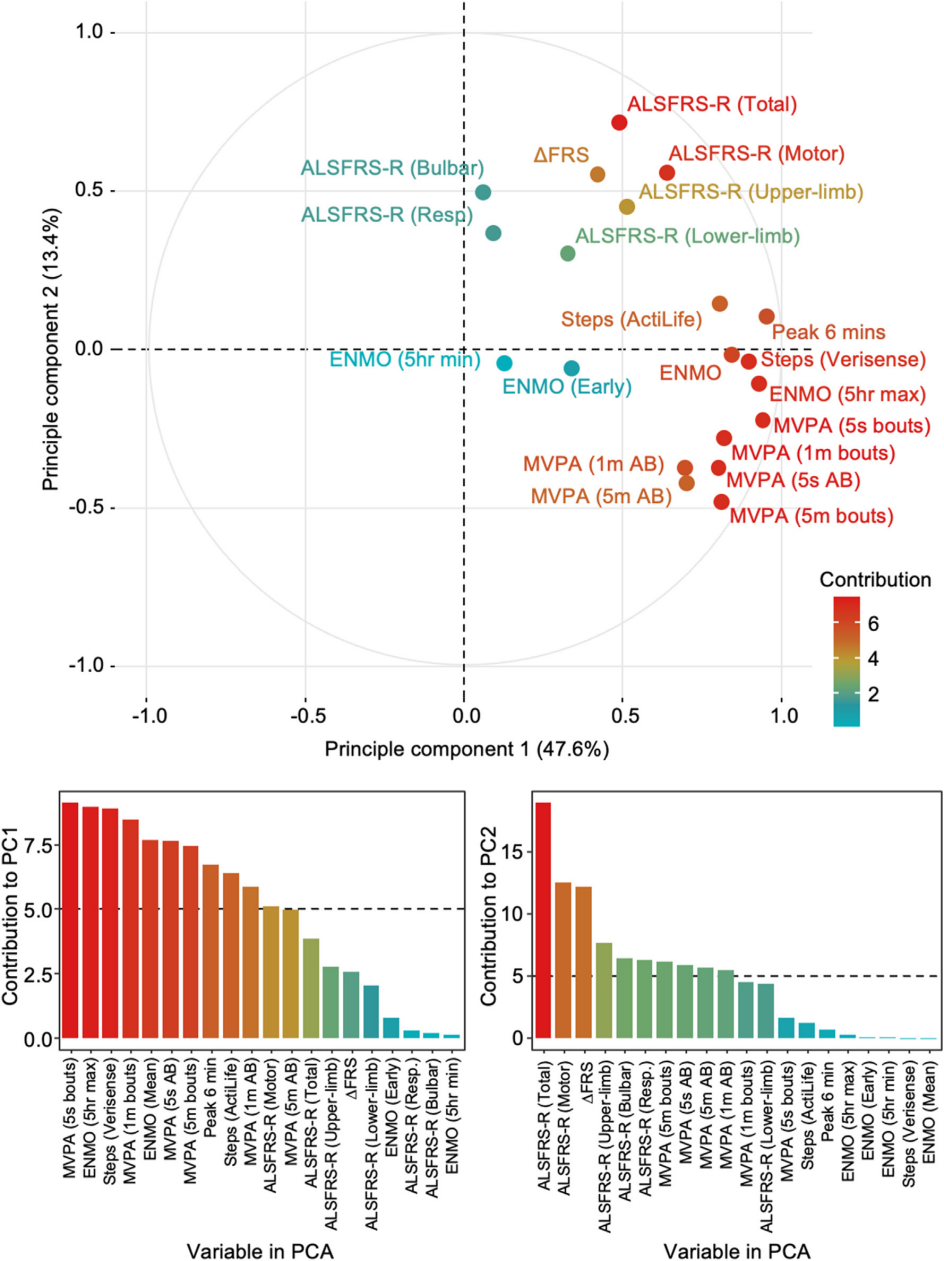
**Principal component analysis of residuals from per-subject ALSFRS-R and accelerometer longitudinal outcomes.** Variables that are a closer together represent more similar longitudinal behaviour. The accelerometer outcomes have different longitudinal dynamics to the ALSFRS-R. Contributions to PC1 and PC2 are highlighted in the plots below. ALSFRS-R, amyotrophic lateral sclerosis functional rating scale—revised; ENMO, Euclidean norm minus one; MVPA, time spent in moderate to vigorous activity.

We next considered whether changes in movement assessed by accelerometry reflected meaningful underlying aspects of the disease, and whether these are distinct from information captured by the ALSFRS-R. We conducted a principal component analysis (PCA) on the per-participant mixed effects residuals (Fig. 4). When considering the full cohort, features derived from accelerometry were found to be more similar to each other than to the ALSFRS-R and were largely associated with their own principal component, supporting their use as a complementary measure. We note also that features derived from accelerometers were mostly unidimensional and largely contribute to principal component 1 (PC1), whereas measures derived from the ALSFRS-R, and specifically the combined motor domain, were multidimensional (Fig. 4). PCA for participants who met TRICALS criteria are presented in Supplemental Figure S3.

### Accelerometer-derived outcomes can be used to power clinical trials

**Fig. 5:**
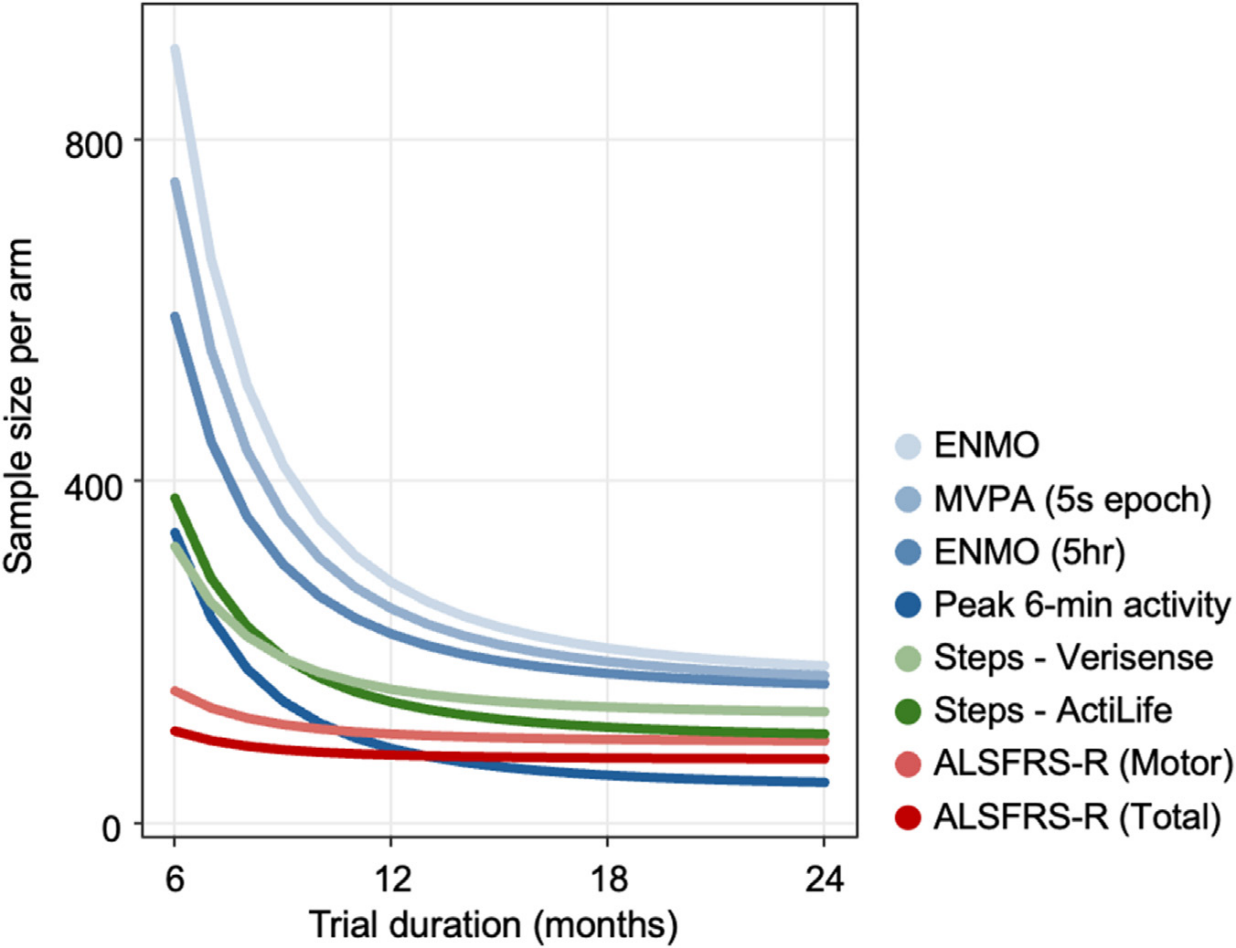
**Predicted per-arm sample size required to detect a 30% reduction in the slope of the target outcome measure, based on 3-monthly assessments.** Predictions assume 80% power, and α = 0.05. ALSFRS-R, amyotrophic lateral sclerosis functional rating scale—revised; ENMO, Euclidean norm minus one; MVPA, time spent in moderate to vigorous activity.

We further explored the potential for passive wearable accelerometer outcomes to monitor clinical trials by limiting our study population to be more representative of clinical trial inclusions, and specifically TRICALS inclusion criteria. Under these conditions, we found an increase (116 vs 103 participants per arm at 12 months) in the number of participants needed to power a study when considering the “motor” ALSFRS-R subdomain scores, relative to the total ALSFRS-R (Fig. 5). Compared to the ALSFFRS-R, all measures of actigraphy performed poorly when considering a short trial duration (i.e. 6 months), however all improved with longer duration trials. Peak 6-min activity showed the most promising outcomes; we note comparable estimates to ALSFRS-R at 12 months (94 vs 102 participants) and smaller trial arm estimates by 18 (66 vs 97 participants) and 24 months (58 vs 95 participants).

### Measures of motor function do not predict survival

**Fig. 6:**
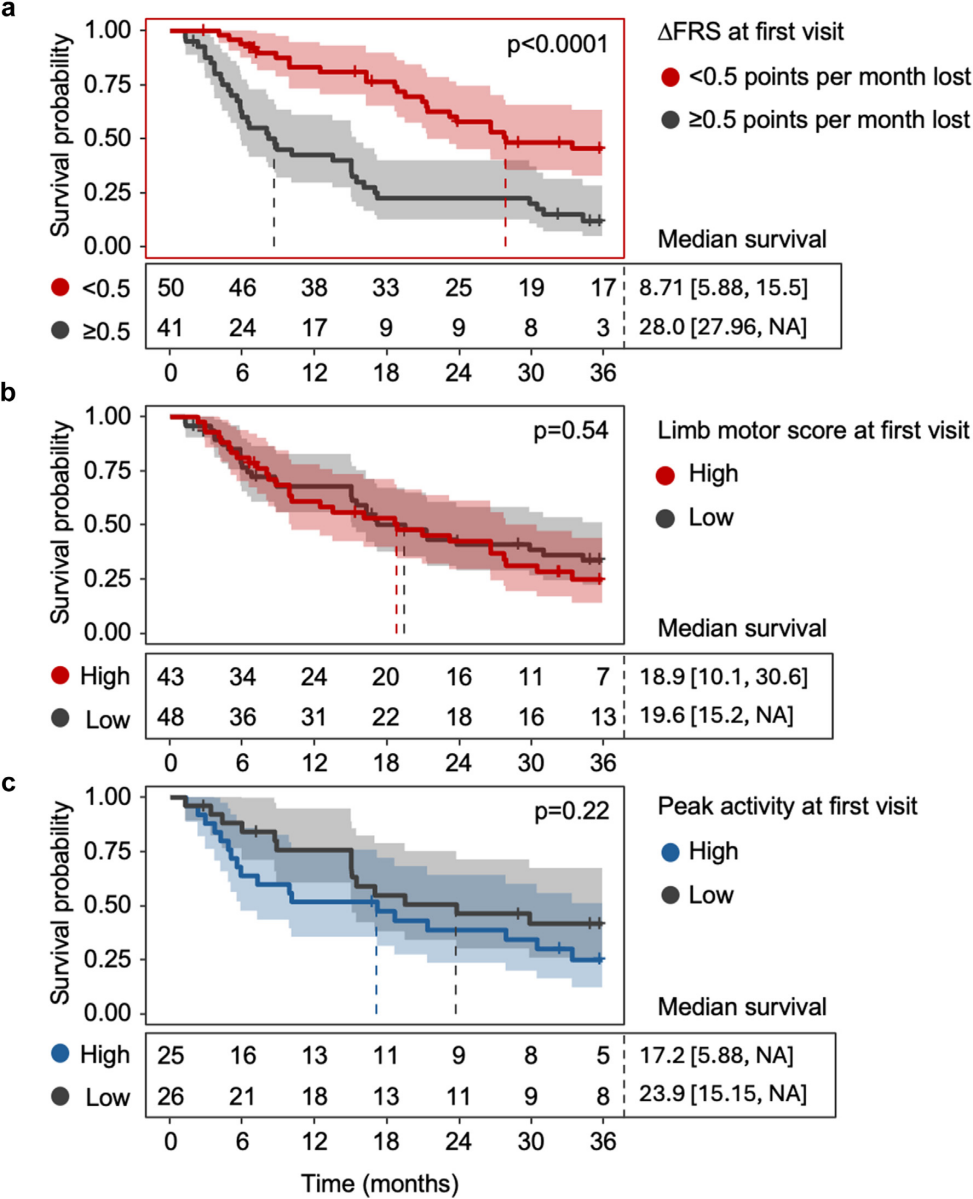
**Kaplan–Meier curves comparing peak activity and ΔFRS. a.** ΔFRS predicts shorter survival. ΔFRS estimated at initial research visit. Measures of **b.** functional capacity as estimated from the ALSFRS-R motor subscore domain, and **c.** peak activity at the initial research visit were not predictive for shorter survival. Curves grouped based on median monthly change estimated from per-participant linear mixed-effects regressions (n = 91). Tables under each graph show number at risk. Not all participants had all measures available at the initial research visit; the presented number at risk reflect this. Hazard ratios (HR) estimated from univariate Cox proportional hazard models. p values estimated from log-rank tests on the Kaplan–Meier curves. Dashed lines indicate median survival times for each group. Shaded area indicates 95% confidence band for survival curve. Numbers in square brackets indicate 95% confidence interval for median survival time. ∗an upper confidence bound was not able to be estimated due to an insufficient number of deaths in this subgroup during the monitoring period. ΔFRS: rate of decline in amyotrophic lateral sclerosis functional rating scale-revised (ASLFRS-R), calculated as [ΔFRS = (ALSFRS-R score–48)/months since symptom onset].

To investigate associations between physical activity and disease outcome, we conducted survival analyses using data from the whole cohort. To capture the relationship between time-varying predictors and risk for death, we used joint models that considered age and sex as covariates (summaries, Table 3; full models presented in Supplementary Table S3). 95 patients were included in the analysis; 61 (64%) passed away during the follow-up period. While the ALSFRS-R total score was predictive for survival (HR = 0.959 [0.926, 0.994]), measures of motor function (including ALSFRS-R motor subscores and measures of actigraphy) were not statistically significantly associated with the hazard. However, the confidence intervals for actigraphy-derived measures (e.g., peak activity HR = 0.985 [0.949, 1.021]) include values consistent with potentially clinically meaningful effects, and smaller associations cannot be ruled out. Kaplan–Meier analyses revealed similar outcomes (Fig. 6). For comparison, we included survival relative to ΔFRS at the time of first study assessment, as this is recognised as being prognostic.

**Table 3:** Summary of hazard ratios estimated from joint models.

	Absolute				Scaled (SD)		
	HR (base units)	95% CI	HR (per 100)	95% CI	HR	95% CI	p
ALSFRS-R (Total)	0.959	[0.926, 0.994]			0.763	[0.609, 0.957]	0.019
ALSFRS-R (Motor)	0.987	[0.943, 1.033]			0.930	[0.750, 1.153]	0.51
Steps (ActiLife)	1.000	[1.000, 1.000]	1.000	[0.995, 1.006]	0.990	[0.733, 1.337]	0.95
Steps (Verisense)	1.000	[1.000, 1.000]	0.998	[0.989, 1.008]	0.937	[0.695, 1.263]	0.67
ENMO (Mean)	1.021	[0.971, 1.072]	4.268	[0.032, 570.7]	1.137	[0.793, 1.631]	0.49
ENMO (5 h peak)	1.001	[0.980, 1.023]	0.986	[0.115, 8.491]	0.998	[0.698, 1.426]	0.99
MVPA (5s epoch)	0.996	[0.983, 1.010]	0.702	[0.180, 2.742]	0.876	[0.566, 1.356]	0.55
Peak 6 min activity	1.000	[0.999, 1.002]	1.041	[0.930, 1.165]	1.085	[0.824, 1.428]	0.56

Hazard ratios estimated from joint models that considered age and sex as covariates for survival, and time as a per-participant random effect for the linear mixed-effects portion of the model. Data shown are hazard ratio [95% confidence interval]. Hazard ratios are reported in their original units, as well as scaled and centred to allow for direct comparability between variables. ALSFRS-R, amyotrophic lateral sclerosis functional rating scale—revised; ENMO, Euclidean norm minus one; MVPA, time spent in moderate to vigorous activity.

## Discussion

We explored the potential of existing passive, wrist-worn accelerometer-derived measures to monitor disease progression in patients with MND, interrogating their value as outcome measures in clinical trial design. As shown previously,^10^ we found that wrist-based metrics decline over time and correlate with conventional indirect assessments of functional capacity, specifically the ALSFRS-R and motor-associated ALSFRS-R subscores. Measures of wrist-based actigraphy capture unique and complementary aspects of disease progression that may not be captured by the ALSFRS-R alone. Furthermore, our data indicate that with careful selection of peak performance metrics, wrist-based outcomes can support the design and conduct of remote clinical trials, achieving comparable samples sizes to ALSFRS-R-based outcomes. The utility of conventional wrist-based measures, however, is currently limited, and most appropriate for use in longer duration trials. Refinement of these clinical outcome measures is needed to fully leverage their potential for shorter-duration studies.

Accelerometry has been routinely used for nearly two decades in large population studies, such as the NHANES.^23^ It is thus surprising that there is no consensus on which specific features should be collected, and how to standardise the reporting and analysis of resulting data, especially in diseases with a high degree of functional decline. Within MND, investigators have explored a wide range of outcomes, including manufacturer-reported step estimates,^24^ power spectral densities of acceleration magnitude,^25^ MET estimates,^7^ acceleration count magnitudes and variabilities,^7^^,^^9^^,^^10^ machine learning-derived performance in task-based assessments,^26^ machine learning-derived regressions trained on high-dimensional “sub-movement” feature sets,^27^ vendor-provided feature sets,^5^ and estimates of upper-limb flexion, extension and rotations.^28^ Each of these approaches highlight important findings, but a direct comparison of more conventional metrics across studies is lacking, limiting the validation of actigraphy as clinical outcome measures in MND. Therefore, our focus was on evaluating a standardised suite of accelerometer-derived outcomes (GGIR), which holds potential for broad clinical application. Our focus was on wrist-based actigraphy, as the wrist is a convenient and established wear-location for continuous, objective monitoring in real-world environments^29^ and remote clinical trials.^30^ Wrist-worn devices serve as versatile platforms capable of capturing multiple outcomes. This wear-location promotes high patient compliance.^31^ Additionally, wrist-based devices have the potential to incorporate physiological measures beyond actigraphy, such as photoplethysmography.

A critical factor in advancing actigraphy as a reliable outcome measure is determining which measures are most sensitive and meaningful in the clinical context. We investigated widely used volumetric measures of physical activity, such as MVPA and step counts. MVPA is an aggregate measure, derived from epoch-level metrics like ENMO, which reflects measure of physical activity intensity^32^ combining these into a broader indicator of moderate to vigorous activity. MVPA generally correlates well with health outcomes,^33^ and has been shown to have a dose–response relationship with mortality.^34^ Despite the popularity of these measures in general health monitoring, our findings suggest that, while offering a reliable index for monitoring progression, MVPA and related measures performed relatively poorly against the conventional use of the ALSFRS-R in clinical trial design. We note, however, that performance by the ALSFRS-R is not optimal over these durations, which raises a broader issue in MND research—existing clinical outcome measures, such as the ALSFRS-R, often require large samples sizes to detect meaningful changes in disease progression, especially in short duration clinical trials.

From our cohort, we estimated that 122 participants are required to detect a 30% reduction in disease progression over 6 months using the ALSFRS-R, a finding consistent with prior studies.^7^ By comparison, over 300 participants are required to achieve similar outcomes using the best performing measures of actigraphy. We found that peak 6-min activity counts, which reflects a maximal value of sustained physical output, may offer a more sensitive measure of actigraphy for optimising study design. When compared directly to motor domains of the ALSFRS-R, peak 6-min activity required smaller trial arms than motor-subscores after 12 months (101 vs 115 participants) to detect a treatment effect. Recent research supports the value of measures of maximal function in tracking neurodegenerative disease progression.^35^ For example, the approval of “stride velocity 95th centile” (SV95C) by the European Medicines Agency for Duchenne muscular dystrophy^35^ underscores its clinical relevance. SV95C, as with the 95th percentile of peak 6-min activity, captures a patient's best physical output, and reduces bias from variations in daily activity.^35^ Similarly, established markers currently used in MND clinical practice, such as forced vital capacity (FVC—a measure of maximal respiratory function) and the ALSFRS-R (a measure of functional capacity during activities of daily living), focus on maximum capabilities in specific functional domains. This suggests that maximal performance measures, which are less influenced by daily routines and external factors, may offer greater sensitivity to change over time, particularly in MND, where disease progression is often highly heterogeneous. Of interest, measures of motor function, including ALSFRS-R motor subscores, did not predict survival, reinforcing that the rate of decline (as seen with ΔFRS), rather than absolute function at a single time point, is more likely to be prognostic. As with other natural history studies,^36^ estimated survival outcomes may not reflect survival in a “typical” clinical cohort. Future comprehensive prospective clinical outcome measure studies should aim to compare typical clinical trial populations with broader observational cohorts to better understand the sensitivity of accelerometer-derived metrics across diverse patient groups, especially when testing assumptions around prognosis.

Principal component analysis shows that ALSFRS-R and actigraphy measures likely capture unique aspects of the impact of disease. Combining such complementary outcome measures is likely to be more effective than relying on a single metric, especially when capturing the complexity of MND progression. The ALS Therapy Development Institute (ALS-TDI) demonstrated that machine learning models trained on multiple accelerometer-derived “sub-movement” features can effectively monitor disease progression in people with ALS,^27^ without relying on the ALSFRS-R as ground truth. Our findings mirror these results, with wrist-based metrics showing moderate correlations with ALSFRS-R scores; their sub-movement features (r = 0.31–0.48)^27^ and our results (r = 0.54–0.66) found comparable correlation strength between the accelerometer-derived wrist outcomes and the ALSFRS-R, supporting the additional use of passive wrist accelerometery for specifically monitoring physical function decline. As with the ALS-TDI study, ours was limited by the absence of a definitive marker of functional decline, raising questions about how well accelerometry-derived outcomes reflect underlying disease progression. Future studies should contrast actigraphy with objective motor function measures, such as the Medical Research Council (MRC) scale or Penn Upper Motor Neuron Score (PUMNS), to better define its role in tracking gross and fine motor function decline.^37^, ^38^, ^39^

Our “clinical trial cohort” of study participants included patients with relatively slow-progressing disease, resulting in a ΔFRS of −0.52, compared to an estimate of −0.98 in a “trial eligible” population.^12^ Thus, while interrogating outcomes in patients who met criteria for clinical trial inclusion–using established TRICALS criteria–our dependence on slower progressing patients may have impacted study outcomes. Beyond these methodological considerations, unmeasured confounders remain a potential limitation. While we adjusted for age and sex in survival models, cognitive decline, anxiety, depression, and variations in daily routines were not explicitly measured, making it difficult to fully isolate motor decline from broader functional changes. The practical application of wrist-worn accelerometry also warrants discussion. We did not assess differences between the dominant and non-dominant limbs, and how different levels of upper-limb muscle weakness may have affected activity. Studies from ALS-TDI suggest that disease severity, rather than limb dominance, is the primary driver of longitudinal actigraphy changes.^27^ In the current study, device placement on the dominant limb may limit generalisability, particularly for individuals with severe upper-limb impairment. Additionally, as we did not compare wrist-based actigraphy to hip-based measures, we cannot comment on the relative value of different wear locations. Future studies should explore optimal placement, though balancing technical accuracy with patient preference may introduce additional complexity. Finally, hazard ratios (HRs) assume proportional hazards over time, which may not always hold in MND.^36^ We accounted for this by employing joint models to incorporate time-varying predictors, though further refinement of statistical approaches is needed.

In conclusion, our results support the use of passive wrist-worn accelerometry for monitoring movement and disease progression in clinical settings and trial design. While conventional measures such as step counts and MVPA may not fully capture the nuances of disease progression, metrics that capture peak performance (in this instance, the 95th percentile of peak 6-min activity) offer promising alternatives. These peak measures provide a clearer view of a patient's maximum functional capacity, which may be more responsive to disease-related changes. Future efforts should prioritise identifying and validating the most effective wearable-derived metrics of maximal function, ensuring that they align with established clinical outcomes. In addition, the statistical properties of accelerometery measures reported here might be improved by longer data collection which can reduce day-to-day variances associated with passive monitoring. SV95C requires several weeks of data collections to achieve the desired performance, significantly longer than more conventional 7 days of actigraphy monitoring. By expanding the array of reliable and sensitive measures and optimising data collection protocol, we can enhance disease monitoring, reduce patient burden through remote assessment, and improve the evaluation of therapeutic interventions in MND.

## Contributors

Study concept and design: CJH, RP, CCG, FJS, STN. Acquisition, follow-up, analysis and interpretation of data: all authors. Draughting of the manuscript: CJH, FJS, STN. Critical revision of the manuscript for important intellectual content: all authors. Administrative and/or material support: FJS, STN. Supervision: FJS, STN. Access to and verification of underlying data: CJH, FJS. All authors read and approved the final version of this manuscript.

## Data sharing statement

Raw data and analytical code are accessible at https://doi.org/10.48610/d75ce7e. Further queries can be directed to the corresponding author (Frederik Steyn, f.steyn@uq.edu.au).

## Declaration of interests

Dr Rakesh Pilkar and Dr Christine C. Guo are employed by ActiGraph LLC, who have a direct interest in the development of actigraphy in research. Their involvement did not affect the conclusions drawn from this research. Dr Nadia Sethi consulted for ALS-TDI, and owns stock in Amgen and AbbVie. The other authors declare no further potential conflicts of interest.
