# Supplementary material for: Identification of passive wrist-worn accelerometry outcomes for improved disease monitoring and trial design in motor neuron disease
Source: eBioMedicine. 2025 May 29;117:105779. doi: 10.1016/j.ebiom.2025.105779 (PMC12166790; doi:10.1016/j.ebiom.2025.105779)
Supplement: Supplementary Tables and Figures [file mmc1.pdf]

Supplementary Tables and Figures:

Supplementary Table 1: Individual questions responses for the ALSFRS-R for all study participants.

	Item	Median [Q1, Q3]
**Question 1**	Speech	3[3,4]
**Question 2**	Salivation	4[3,4]
**Question 3**	Swallowing	4[3,4]
**Question 4**	Handwriting	3[3,4]
**Question 5a**	Cutting food and handling utensils, no gastrotomy	3[2,4]
**Question 5b**	Cutting food and handling utensils, gastrotomy	4[4,4]
**Question 6**	Dressing and hygiene	3[2,3]
**Question 7**	Turning in bed and adjusting bed clothes	3[3,4]
**Question 8**	Walking	2[2,4]
**Question 9**	Climbing stairs	1[1,3]
**Question 10**	Difficulty breathing	4[3,4]
**Question 11**	Difficulty breathing when lying flat	4[4,4]
**Question 12**	Respiratory insufficiency	4[4,4]

Data are presented as median with 1st [Q1] and 3rd [Q3] quartiles. ALSFRS-R: amyotrophic lateral sclerosis functional rating scale. For 5B, one participant was dependent on gastrotomy at their first research visit.

Supplementary Table 2. Overview of accelerometer-derived outcomes considered for study inclusion.

Source	Parameter	Description
GGIR	ENMO (5hr min)	Mean ENMO during 5 **least** active hours in the day (determined by rollling mean)
	ENMO (5hr max)	Mean ENMO during 5 **most** active hours in the day (determined by rollling mean)
	ENMO (1-6am)	Mean ENMO between 1am and 6am
	ENMO	Mean ENMO
	MVPA (5-sec epoch)	Mean MVPA with 5-second epoch
	MVPA (1-min epoch)	Mean MVPA with 1-minute epoch
	MVPA (5-min epoch)	Mean MVPA with 5-minute epoch
	MVPA (1-min bouts)	Mean MVPA with 5-second epoch within highly active 1-min bouts (MVPA > 80%)
	MVPA (5-min bouts)	Mean MVPA with 5-second epoch within highly active 5-min bouts (MVPA > 80%)
	MVPA (10-min bouts)	Mean MVPA with 5-second epoch within highly active 10-min bouts (MVPA > 80%)
Verisense	Steps (Verisense)	Step counts determined by Verisense algorithm
Actilife	Steps (ActiLife)	Step counts reported by ActiLife software
	Peak 6min activity	95th percentile of ActiGraph "counts" within a rolling 6 mins window

ENMO: Euclidean norm minus one; MVPA: time spent in medium-to-vigorous physical activity.

Supplementary Table 3. Longitudinal performance of each accelerometer-derived outcome.

Source	Parameter	Monthly change	SD/Month	ALSFRS-R Correlation	Motor Correlation
GGIR	ENMO (5hr min)	0.03 [-0.020, 0.080]	0.022 [-0.013, 0.056]	-0.009 [-0.166, 0.148]	0.037 [-0.120, 0.193]
	ENMO (5hr max)	-0.714 [-1.173, -0.256]	-0.043 [-0.071, -0.015]	0.507 [0.380, 0.615]	0.608 [0.499, 0.699]
	ENMO (1-6am)	0.014 [-0.049, 0.076]	0.004 [-0.013, 0.021]	0.104 [-0.054, 0.257]	0.187 [0.031, 0.334]
	ENMO	-0.279 [-0.500, -0.059]	-0.038 [-0.068, -0.008]	0.471 [0.339, 0.585]	0.613 [0.504, 0.702]
	MVPA (5-sec epoch)	-1.225 [-1.899, -0.550]	-0.04 [-0.062, -0.018]	0.472 [0.340, 0.585]	0.545 [0.424, 0.646]
	MVPA (1-min epoch)	-0.781 [-1.230, -0.333]	-0.032 [-0.050, -0.014]	0.416 [0.277, 0.538]	0.461 [0.328, 0.576]
	MVPA (5-min epoch)	-0.397 [-0.605, -0.189]	-0.025 [-0.043, -0.008]	0.377 [0.234, 0.504]	0.396 [0.254, 0.520]
	MVPA (1-min bouts)	-0.38 [-0.628, -0.132]	-0.025 [-0.041, -0.009]	0.408 [0.268, 0.531]	0.417 [0.278, 0.539]
	MVPA (5-min bouts)	-0.301 [-0.412, -0.190]	-0.028 [-0.038, -0.019]	0.368 [0.224, 0.497]	0.355 [0.209, 0.485]
	MVPA (10-min bouts)	-0.243 [-0.359, -0.126]	-0.013 [-0.020, -0.007]	0.337 [0.190, 0.469]	0.319 [0.170, 0.453]
Verisense	Steps (Verisense)	-166 [-251, -82]	-0.053 [-0.079, -0.026]	0.520 [0.395, 0.626]	0.658 [0.558, 0.739]
Actilife	Steps (ActiLife)	-353 [-353, -353]	-0.059 [-0.089, -0.030]	0.510 [0.384, 0.618]	0.678 [0.583, 0.755]
	Peak 6min activity	-16.1 [-21.7, -10.5]	-0.066 [-0.089, -0.043]	0.552 [0.432, 0.652]	0.676 [0.581, 0.753]

Monthly declines and correlations with ALSFRS-R were estimated from linear mixed-effects regressions, with a participant-wise random intercept and slope. Data are presented as estimate [95% confidence interval]. ALSFRS-R: amyotrophic lateral sclerosis functional rating scale – revised; ENMO: Euclidean norm minus one; MVPA: time spent in moderate to vigorous activity.

Supplementary Figures:

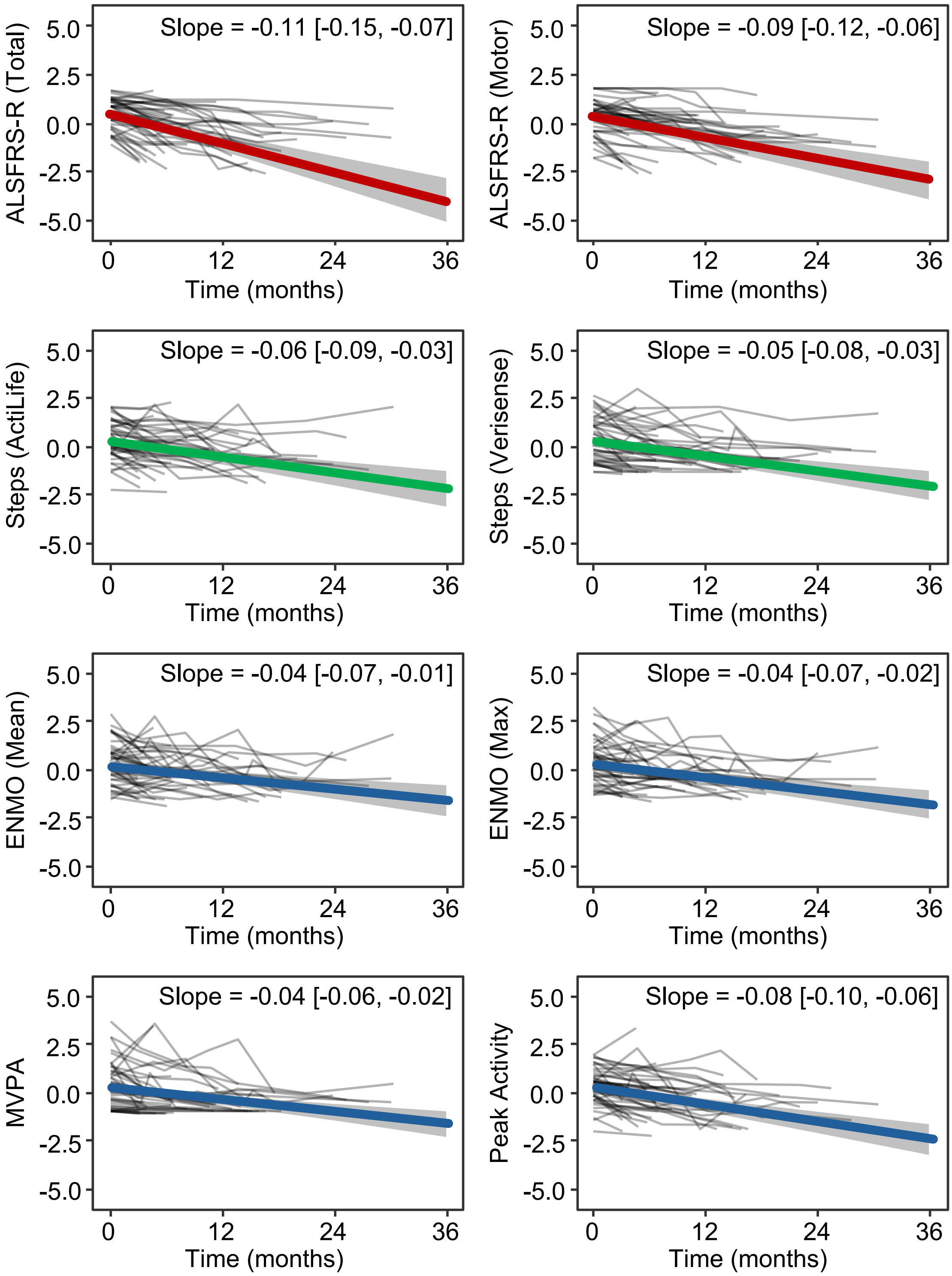

Supplementary Figure 1. Spaghetti plots of accelerometer-derived outcomes and ALSFRS-R over time, limited to the trial-eligible cohort. Outcomes are standardised to standard deviations from the mean (n=65). Black lines show per-participant individual traces over time, and coloured lines shows the "population" trend. Coloured trendline estimated from the mixed-effects component of a joint model, with a random effect for time and participant. Age and sex were considered covariates for survival. 95% confidence interval of the estimate is shaded in grey. All estimates are p<0.0001. ALSFRS-R: amyotrophic lateral sclerosis functional rating scale – revised; ENMO: Euclidean norm minus one; MVPA: time spent in moderate to vigorous activity.

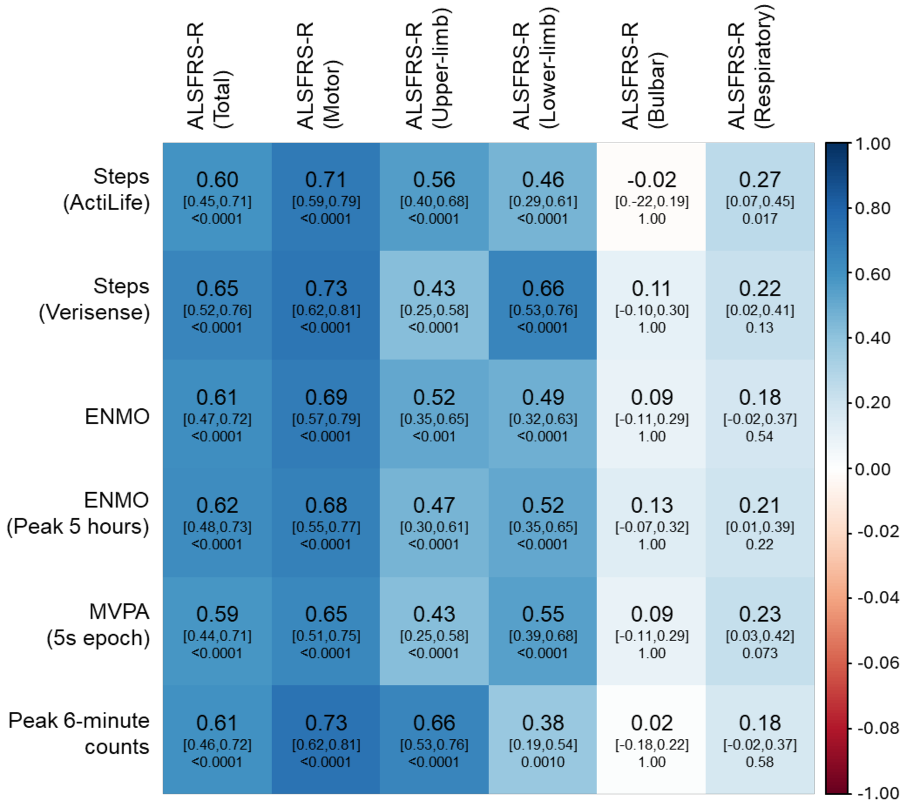

Supplementary Figure 2. Correlations were estimated from the per-participant residuals from a joint model, with regressions fit to each parameter over time to control for time as a confounding factor (n=65). Data is limited to participants meeting the TRICALS clinical trial criteria. Age and sex were considered covariates for survival. Correlation strengths are shown within the matrix as numeric and colour. Main numbers show estimated correlation coefficients; subscripts show p values. ALSFRS-R: amyotrophic lateral sclerosis functional rating scale – revised; ENMO: Euclidean norm minus one; MVPA: time spent in moderate to vigorous activity.

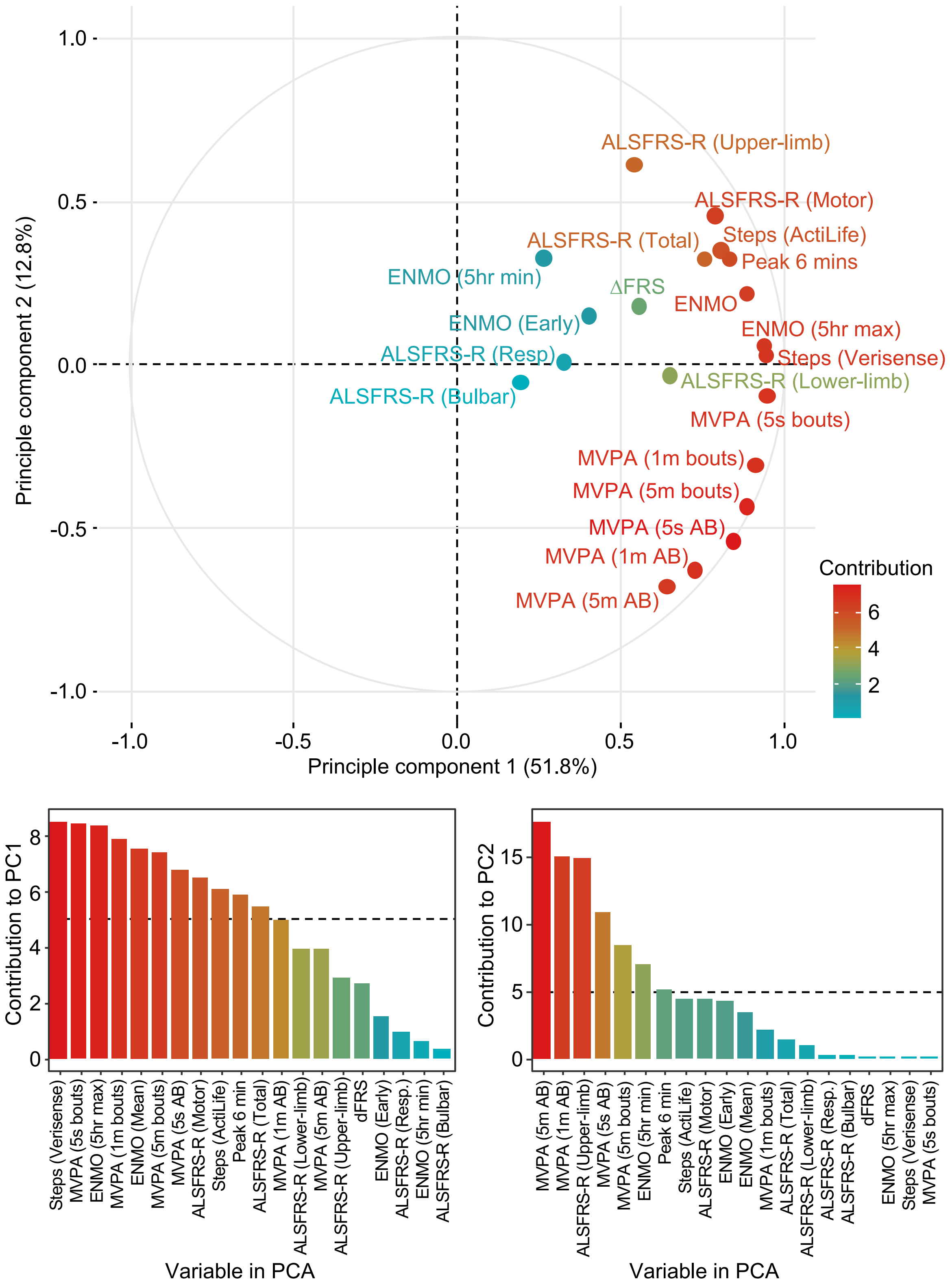

Supplementary Figure 3. Principle components analysis of residuals from per-subject ALSFRS-R and accelerometer longitudinal outcomes based on the trial-eligible cohort. Variables that are a closer together represent more similar longitudinal behaviour. The accelerometer outcomes have different longitudinal dynamics to the ALSFRS-R. Contributions to PC1 and PC2 are highlighted in the plots below. ALSFRS-R: amyotrophic lateral sclerosis functional rating scale – revised; ENMO: Euclidean norm minus one; MVPA: time spent in moderate to vigorous activity.
